# Accuracy of temporomandibular disorders diagnosis evaluated through the diagnostic criteria for temporomandibular disorder (DC/TDM) Axis II compared to the Axis I evaluations: a systematic review and meta-analysis

**DOI:** 10.1186/s12903-024-03983-7

**Published:** 2024-03-02

**Authors:** Giuseppe Minervini, Maria Maddalena Marrapodi, Yuliia Siurkel, Marco Cicciù, Vincenzo Ronsivalle

**Affiliations:** 1 Saveetha Dental College and Hospitals, Saveetha Institute of Medical and Technical Sciences (SIMATS), Saveetha University, Chennai, Tamil Nadu India; 2 Multidisciplinary Department of Medical-Surgical and Dental Specialties, University of Campania, Luigi Vanvitelli, Caserta, 81100 Italy; 3 Department of Woman, Child and General and Specialist Surgery, University of Campania, Luigi Vanvitelli, Naples, 80121 Italy; 4 International European University School of Medicine, Akademika Hlushkova Ave, 42В, Kyiv, 03187 Ukraine; 5 Department of Biomedical and Surgical and Biomedical Sciences, Catania University, Catania, 95123 Italy

**Keywords:** Axis I, Axis II, Temporomandibular disorders, DC/TMD

## Abstract

**Background:**

The temporomandibular joint (TMJ) is a complex joint that facilitates mandibular movements during speech, chewing, and swallowing activities. The Axis I evaluation of the DC/TMD focuses on assessing physical diagnoses related to TMDs. It includes an assessment of pain and functional limitations, such as jaw opening range, joint sounds, and joint tenderness. The Axis II evaluation of the DC/TMD provides information on the patient’s psychological status and quality of life. This Systematic Review with Meta-Analysis aimed to evaluate the accuracy of Temporomandibular Disorders diagnosis considered through the Diagnostic Criteria for Temporomandibular Disorder (DC/TDM) axis II compared to the Axis I evaluations.

**Methods:**

A search was made in PubMed, Web of Science and Lilacs for articles published from the inception until 20 January 2023. We applied the Population, Exposure, Comparator, and Outcomes (PECO) model [1] to assess document eligibility. Only studies that evaluated patients by DC/TMD Axis I and Axis II were considered. Review Manager version 5.2.8 (Cochrane Collaboration) was used for the pooled analysis. We measured the odds ratio (OR) between the two groups (Axis I and Axis II).

**Results:**

Fifty-one articles were selected because of the search. Four papers were excluded before the screening: 2 pieces were not in English, and two were reviewed. The remaining 47 articles were selected for the title and abstract screening to evaluate whether they met the PECO criteria. Among these, four papers were established; the overall effect showed that there was no difference in TMD diagnosis between Axis I and Axis II (RR 1.17; 95% CI: 0.80– 1.71; Z:0.82; *P* = .41), suggesting that there is no difference between Axis I and Axis II.

**Conclusion:**

In conclusion, DC/TMD is an effective tool for the diagnosis of TMD. It improves the accuracy of TMD diagnosis, allows for the classification of subtypes, and assesses psychosocial factors that may impact the development or maintenance of TMD symptoms.

## Introduction

The temporomandibular joint (TMJ) is a complex joint that facilitates mandibular movements during speech, chewing, and swallowing activities. Temporomandibular disorders (TMDs) affect the TMJ and its associated muscles and structures, resulting in pain, dysfunction, and other symptoms. Diagnosing TMDs can be challenging due to their varied etiologies and clinical presentations [2, 3].

To address this challenge, the Research Diagnostic Criteria for Temporomandibular Disorders (RDC/TMD) was developed in the early 1990s. Since then, the diagnostic criteria have been updated, and the Diagnostic Criteria for Temporomandibular Disorders (DC/TMD) were published in 2014. The DC/TMD is a comprehensive, standardized diagnostic tool that includes both an Axis I and an Axis II evaluation [4–13].

The Axis I evaluation of the DC/TMD focuses on assessing physical diagnoses related to TMDs. It includes an assessment of pain and functional limitations, such as jaw opening range, joint sounds, and joint tenderness. Additionally, it consists of evaluating parafunctional habits, such as clenching and grinding, and occlusal factors, such as dental malocclusion [14–21].

In contrast, the Axis II evaluation of the DC/TMD assesses the psychosocial aspects of TMDs, such as anxiety, depression, and quality of life. The Axis II evaluation includes self-report questionnaires and interviews that evaluate the patient’s emotional status, pain-related disability and the impact of the TMD on their daily activities [22–33].

The DC/TMD has been widely accepted and implemented in clinical and research settings to improve the accuracy and consistency of TMD diagnosis. By including both Axis I and Axis II evaluations, the DC/TMD allows for a comprehensive review of the patient’s condition, leading to a more personalized and effective treatment approach.

The DC/TMD has been shown to have high validity and reliability in various populations, including children, adults, and older adults. It has also been used in studies investigating the prevalence and risk factors of TMDs and the effectiveness of various treatment modalities.

One of the key differences between the Axis I and Axis II evaluations is that the Axis I evaluation focuses on physical diagnoses. In contrast, the Axis II evaluation focuses on psychosocial factors. This reflects the multifactorial nature of TMDs, often influenced by physical and psychological factors [34].

The Axis I evaluation of the DC/TMD provides essential information on the nature and severity of the patient’s physical symptoms. This information can help guide the selection of appropriate treatment modalities, such as medication, physical therapy, or splint therapy. The Axis I evaluation can also help identify any underlying physical conditions contributing to the patient’s TMD symptoms, such as arthritis or joint degeneration.

The Axis II evaluation of the DC/TMD provides information on the patient’s psychological status and quality of life. This information can identify any underlying psychosocial factors contributing to the patient’s TMD symptoms, such as anxiety, depression, or stress. Addressing these psychosocial factors can help improve the patient’s overall well-being and response to treatment [35].

In conclusion, the DC/TMD is a comprehensive and standardized diagnostic tool that includes both an Axis I and an Axis II evaluation. The DC/TMD has been widely accepted and implemented in clinical and research settings and has been shown to have high validity and reliability. The Axis I and Axis II evaluations provide essential information on the physical and psychosocial aspects of TMDs, respectively, allowing for a more personalized and effective treatment approach [36].

This Systematic Review with Meta-Analysis aimed to evaluate the accuracy of Temporomandibular Disorders diagnosis considered through the Diagnostic Criteria for Temporomandibular Disorder (DC/TDM) axis II compared to the Axis I evaluations.

## Materials and methods

### Eligibility criteria

The PECO is the following:

- P) Participants consisted of the population.
- E) The Exposure consisted of Temporomandibular Disorders.
- C) The Comparison consisted of Temporomandibular Disorders diagnosis evaluated through the Diagnostic Criteria for Temporomandibular Disorder (DC/TDM) axis II compared to the Axis I evaluations.
- O) The Outcome consisted of the Accuracy of Temporomandibular Disorders diagnosis evaluated through the Diagnostic Criteria for Temporomandibular Disorder (DC/TDM) axis II compared to the Axis I evaluations.

### Search strategy

The Search were made in PubMed, Web of Science and Scopus until May 1, 2023. Table 1 reports the complete keywords used for the search.

**Table 1:** Search strategy

**PubMed** Search: (DIAGNOSTIC CRITERIA FOR TEMPOROMANDIBULAR DISORDERS (DC/TMD)) AND (AXIS II) ((“diagnosis“[MeSH Terms] OR “diagnosis“[All Fields] OR “diagnostic“[All Fields] OR “diagnostical“[All Fields] OR “diagnostically“[All Fields] OR “diagnostics“[All Fields]) AND (“criteria s“[All Fields] OR “criterias“[All Fields] OR “standards“[MeSH Subheading] OR “standards“[All Fields] OR “criteria“[All Fields]) AND (“temporomandibular joint disorders“[MeSH Terms] OR (“temporomandibular“[All Fields] AND “joint“[All Fields] AND “disorders“[All Fields]) OR “temporomandibular joint disorders“[All Fields] OR (“temporomandibular“[All Fields] AND “disorders“[All Fields]) OR “temporomandibular disorders“[All Fields])) AND “dc tmd“[All Fields] AND ((“axis, cervical vertebra“[MeSH Terms] OR (“axis“[All Fields] AND “cervical“[All Fields] AND “vertebra“[All Fields]) OR “cervical vertebra axis“[All Fields] OR “axis“[All Fields]) AND “II“[All Fields])
**Scopus** TITLE-ABS-KEY (diagnostic AND criteria AND for AND temporomandibular AND disorder AND dc/tmd AND axis AND ii)
**Lilacs** diagnostic criteria for temporomandibular disorders dc/tmd [Palavras] and axis II [Palavras]

The systematic review has been registered in PROSPERO with CRD42022327470.

### Data extraction

The following data were extracted: (1) First author; (2) Year of publication; (3) Nationality; (4) Number of study participants (5) Age of study participants (case vs. controls); (6) Diagnostic criteria/tools used for the diagnosis of TMD; (7) Correlation between Axis I and II (8) Significance of the study.

### Quality assessment

The bias were evaluated using RoB2 by Cochrane. Two reviewers evaluated the possible bias using six domains.

### Statistical analysis

The statistical analysis was performed using Review Manager version 5.2.8. We measured the difference between Axis I and Axis II.

## Results

### Study characteristics

**Fig. 1:**
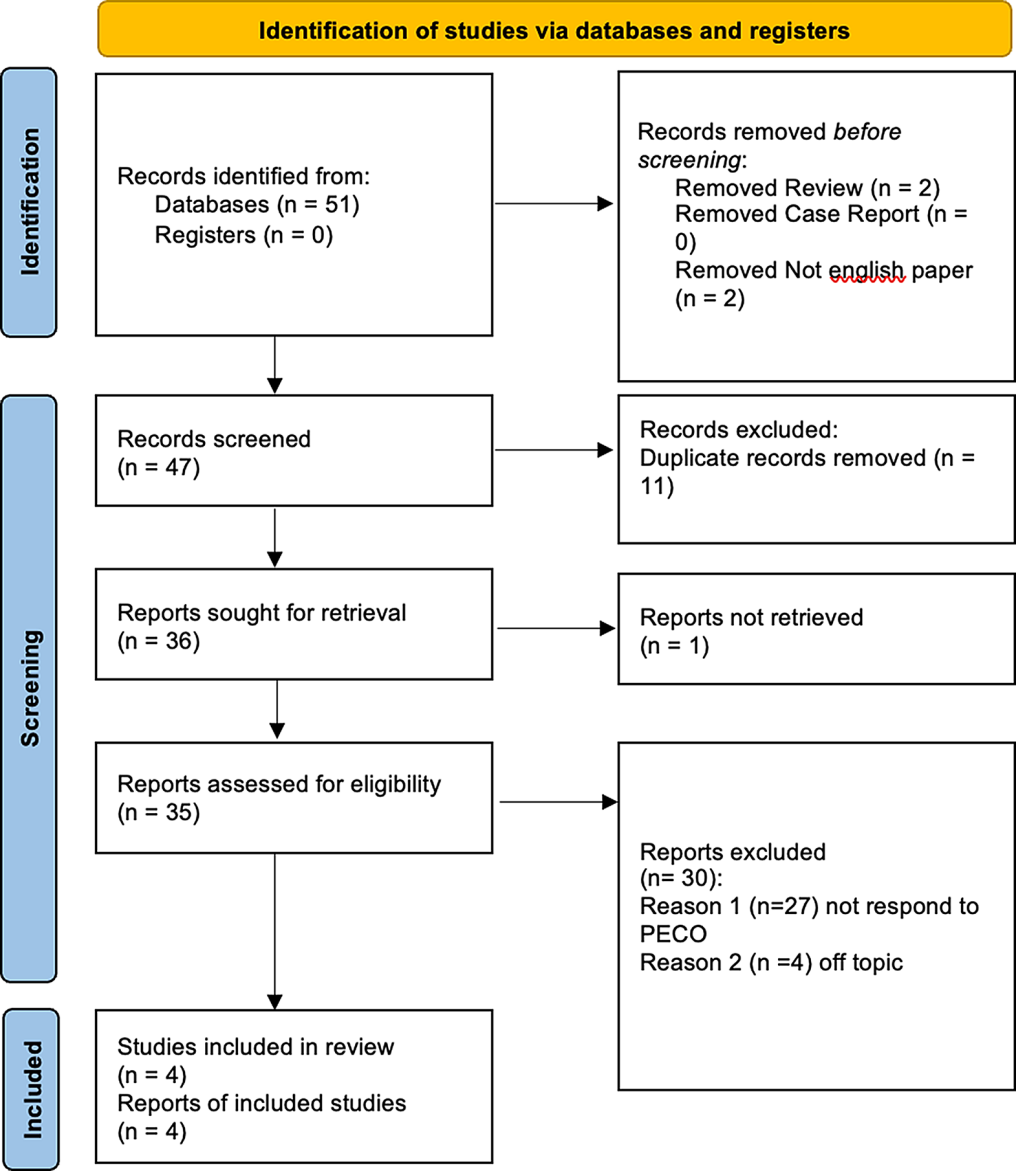
From: Page MJ, McKenzie JE, Bossuyt PM, Boutron I, Hoffmann TC, Mulrow CD, et al. The PRISMA 2020 statement: an updated guideline for reporting systematic reviews. BMJ 2021;372:n71. doi: 10.1136/bmj.n71

Four studies were included in the systematic review and were considered for the metanalysis, as illustrated in the PRISMA 2020 flowchart in Fig. 1. The included studies have been published between 2022 and 2023. The four included studies were Prospective studies in design or retrospective studies. All of these studies compare the effectiveness of DC/TMD Axis I with DC/TMD Axis II to assess how they can be interchangeable in some cases and how TMDs are caused not only by physiological issues but also by a psycho-social component. The data extracted from each study, as reported in the paragraph “data extraction”.

### Main findings

The included subjects in this review were 773. The ‘average age of the study participants is 35 years old. All patients were evaluated and underwent DC/TMD Axis I, followed by Axis II.

Alrashdan evaluated and compared Axis I with Axis II. By the Axis I DC/TMD procedure, 98 TMD patients had their pain-related disabilities, psychological distress, and stress reactivity evaluated. One third of patients (32%) had high levels of pain-related impairment, and just over half of patients (49%) had high levels of distinctive pain (self-reported TMJ-related pain). Furthermore, most patients (41% and 39%) reported moderate to severe distress and stress reactivity. The Graded Chronic Pain Scale (GCPS) and the pain-related TMD subgroups were significantly correlated [37]. Winocur-Arias compared the effectiveness of DC/TMD in diagnosing local myalgia and myofascial pain using Axis I and Axis II. All consecutive TMD patients who received a DC/TMD diagnosis at our facility between 2015 and 2018 were included in this retrospective analysis. Patients with local myalgia and myofascial pain with referral were compared regarding their Axis I and II results. Statistical significance was defined as a p-value of 0.05. The study involved 255 patients, 114 with local myalgia and 83 with myofascial pain with referral, with a mean age of 37.8 15.34 years. In the latter group, there were significantly greater levels of sadness, non-specific physical symptoms, headaches attributed to TMD (HAattrTMD), and characteristic pain intensity (CPI) [38]. Reiter’s study compared the effectiveness of diagnosing TMD headaches using Axis I and II. This retrospective analysis included 220 patients with painful TMD—60 with and 160 without HAattrTMD—. The patients’ Axis I and II scores were compared using the Diagnostic Criteria for TMD (DC/TMD). Statistical significance was defined as a P value of 0.05. Results: A diagnosis of HAattrTMD was given to 27.3% of the patients. Local myalgia was significantly more prevalent in the non-HAattrTMD group (P 0.001), but myofascial pain with referral was much more commonplace in the HAattrTMD group (P 0.001). The HAattrTMD group had significantly greater levels of depressive symptoms (*P* = .002), nonspecific physical symptoms (*P* = .004), graded chronic pain (*P* = .008), and pain catastrophizing (*P* = .013), as well as characteristic pain intensity (*P* = .003). HAattrTMD was positively correlated with non-specific physical symptoms (odds ratio [OR] = 1.098, 95% CI = 1.006 to 1.200, *P* = .037). A negative correlation existed between local myalgia and HAattrTMD (OR = 0.295, 95% CI = 0.098 to 0.887, *P* = .030) [39]. Yildiz’s study aims to assess the frequency of DC / TMD diagnoses among people with internal TMJ derangements. Two hundred adults over 18 with internal derangements. Axis I and II of the Diagnostic Criteria for Temporomandibular Disorders (DC / TMD) were used. There were 3.6 times as many female patients as male patients (156 versus 44). The likelihood of internal TMJ derangement in the right or left TMJ did not significantly correlate with gender, age, educational attainment, marital status, or occupation (*p* > .05).TMJ internal disorder and Axis II scores correlate significantly (p 0.05). Left TMJ internal dysfunction significantly correlates with GAD 7 and PHQ 4 scores (p 0.05) [40]. All results are represented in the Table 2.

**Table 2:** Principal elements of the studies which formed part of the present systematic analysis

Author	Year	Nationality	Number of case vs. control	Age	Diagnostic tool of TMD	Correlation between Axis I and II	Significance of study
Alrashdan	2022	Jordan	98 patients/49 patients	32.7 years	DC/TMD Axis I DC/TMD Axis II	Pain related TMD r 0.312 0.002* IAD *r* − .309 0.002* Headeche r 0.317 0.001*	Association between clinical symptoms and psychosocial status
Winocur-Arias	2022	Israel	255 patients/66 patients	37 yrs	DC/TMD Axis I DC/TMD Axis II	Myofascial pain. With referral 6.38 ± 5.61 6.00 (2.00–8.00) P:0.033*	Myofascial pain with referral is associated and correlated with increased depressive state and thus with Axis II
Reiter	2021	Israel	220 patients/60 patients	37.8 yrs	DC/TMD Axis I DC/TMD Axis II	Patients with HAattrTMD Evaluated in axis I and II (*P* = .008)*	HAattrTMD group had significantly higher levels of depression
Yildiz	2023	Turkey	200 patients/507 patients	28.46	DC/TMD Axis I DC/TMD Axis II	TMJ internal disorders association with Axis II 0.206*	The results of DC / TMD Axis I are compared with Axis II

*Statistically significant correlation

### Meta-analysis

The included studies had a high heterogeneity (*I*2 = 84%). Therefore the meta-analysis was conducted by random model effect. We considered as an outcome the difference in the diagnosis of TMD between Axis I and Axis II.

**Fig. 2:**
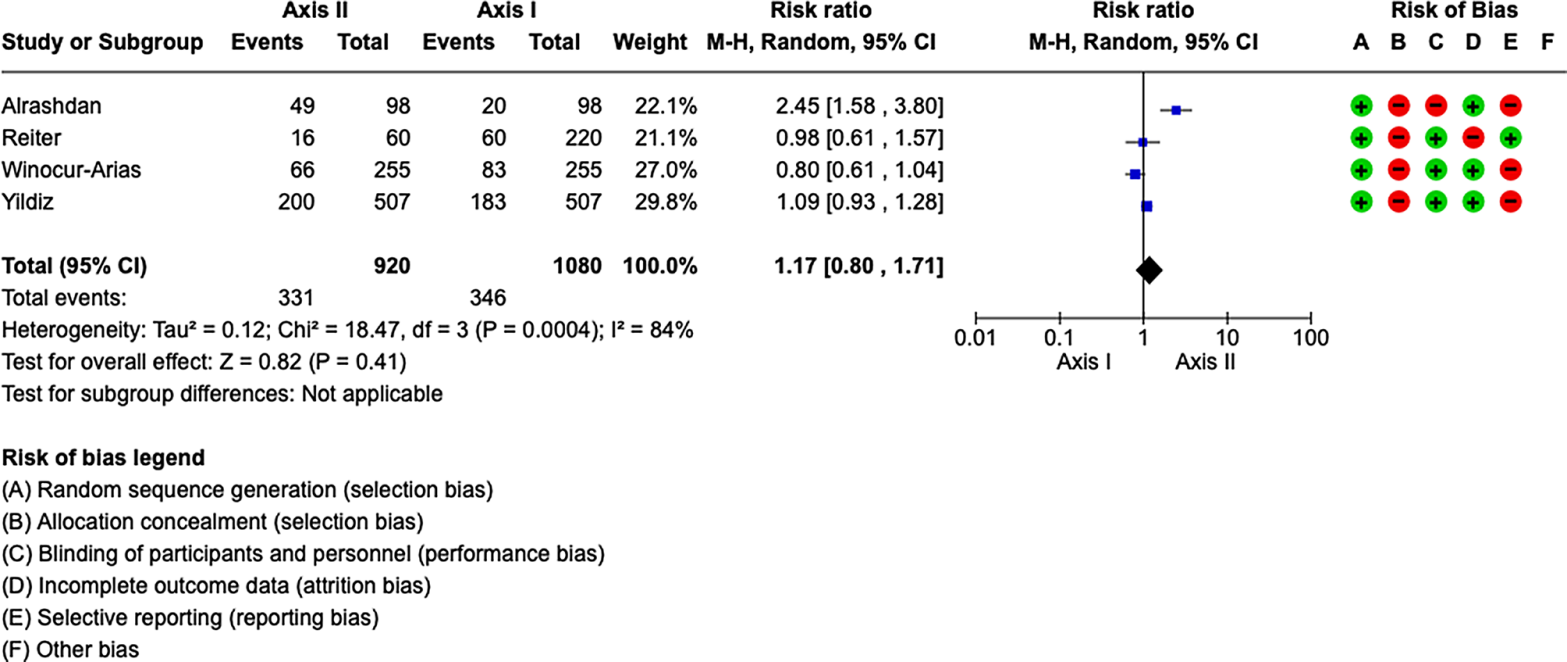
Forest plot of the studies included in this meta-analysis

The overall effect, reported in the forest plot (Fig. 2), showed that there was no difference in In the accuracy between Axis I and Axis II for the diagnosis of TMD (RR 1.17; 95% CI: 0.80– 1.71; Z:0.82; *P* = .41), suggesting that there is no difference between Axis I and Axis II.

### Quality assessment and risk of bias

**Fig. 3:**
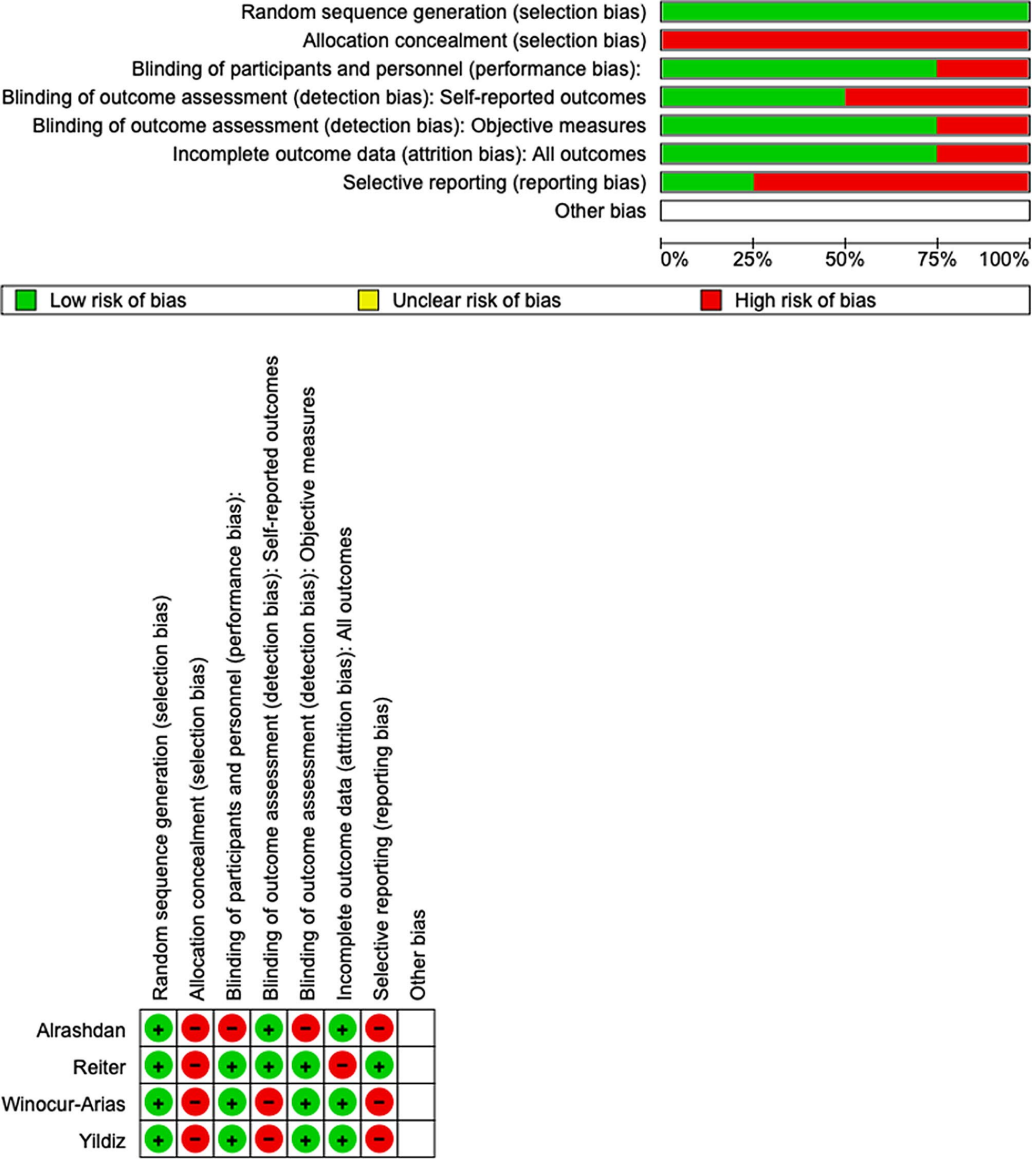
Risk of bias summary

The risk of bias in the included studies was reported in Fig. 3. Regarding the randomization process, all studies had a low risk of bias, and allocation concealment had a high chance. Only three studies excluded a performance; two studies ensured an increased risk of detection bias (self-reported outcomes), and 3 of the included studies present low detection bias (Fig. 3).

## Discussion

Temporomandibular disorders (TMD) are a group of conditions affecting the temporomandibular joint (TMJ) and surrounding structures, which can cause pain and dysfunction. The Diagnostic Criteria for Temporomandibular Disorders (DC/TMD) is a comprehensive tool developed to aid in diagnosing TMD. This scientific discussion will describe the use of DC/TMD in temporomandibular diagnosis and discuss the differences between the Axis I and Axis II evaluations [41].

A collaboration of international experts developed DC/TMD, and is currently the most widely used and accepted tool for diagnosing TMD. The diagnostic criteria are organized into two axes: Axis I evaluates the physical examination and clinical history. In contrast, Axis II considers psychosocial factors that may contribute to the development or maintenance of TMD [42–44].

The Axis I evaluation includes the patient’s physical examination and clinical history. The physical examination involves assessing the patient’s TMJ, masticatory muscles, and occlusion. Clinical history includes collecting information on the patient’s pain and function, including the duration, intensity, and location of pain and any limitations in jaw movement [45].

The Axis II evaluation assesses psychosocial factors that may influence the development or maintenance of TMD. This includes collecting information on the patient’s anxiety, depression, pain catastrophizing, and quality of life. This evaluation helps identify any psychosocial factors that may require treatment alongside the physical symptoms of TMD.

The use of DC/TMD has several benefits, including standardized diagnostic criteria, improved communication between healthcare professionals, and improved patient management. DC/TMD can aid in accurate diagnosis and treatment planning, improving patient outcomes [46].

In conclusion, DC/TMD is a comprehensive tool for diagnosing TMD. Axis I and II evaluations provide a thorough assessment of the patient’s physical symptoms and psychosocial factors. Implementing DC/TMD can aid in accurately diagnosing and treating TMD, leading to improved patient outcomes [47].

Furthermore, there is a need for continued research to validate the tool’s effectiveness and improve its utility. More studies are needed to assess the reliability and validity of DC/TMD in different populations, such as children and elderly patients, and to investigate its sensitivity and specificity for diagnosing TMD [48, 49].

In conclusion, the DC/TMD is a comprehensive tool that aids in accurately diagnosing and treating TMD by assessing physical and psychosocial factors. While its use has some limitations, its benefits make it an essential tool in diagnosing and managing TMD. Ongoing research is necessary to refine and validate the device, leading to better patient outcomes in the future.

Several studies have evaluated the effectiveness of DC/TMD in diagnosing TMD [50, 51]. A systematic review of 38 studies found that DC/TMD had a high diagnostic accuracy in identifying patients with TMD, with a sensitivity of 0.85 and a specificity of 0.80 [52]. Another study found that using DC/TMD improved the accuracy of TMD diagnosis and reduced misclassification rates compared to non-standardized diagnostic criteria.

In addition to improving the accuracy of TMD diagnosis, DC/TMD has also led to a better understanding of the different subtypes of TMD. Axis I evaluation assesses physical factors, including joint or muscle pain, jaw dysfunction, and malocclusion. Based on the results of Axis I evaluation, patients can be classified into subtypes, including muscle disorder, joint disorder, or a combination of both. This classification system allows for a more targeted and effective treatment plan for each subtype of TMD [53].

Axis II evaluation assesses psychosocial factors, such as anxiety, depression, and pain catastrophizing. These factors may impact the development, progression, or maintenance of TMD symptoms. By including Axis II evaluation in TMD diagnosis, healthcare professionals can develop a comprehensive treatment plan that addresses physical and psychosocial factors. This approach has improved patient outcomes, such as reduced pain and improved quality of life [54, 55]. Effective management of TMDs requires an integrated approach that considers both Axis 1 and Axis 2 findings. Treatments may include:

Physical Therapies: Such as physical therapy exercises, massage, and the application of heat or cold.

Dental Interventions: Including occlusal appliances (mouthguards) to reduce clenching and grinding, and corrective dental treatments to address bite abnormalities.

Medications: Pain relievers, muscle relaxants, or anti-inflammatory drugs to manage symptoms.

Behavioral Therapies: Cognitive-behavioral therapy (CBT) and stress management techniques to address psychological and behavioral factors.

Education and Self-care: Teaching patients about their condition and strategies to manage symptoms, such as relaxation techniques and posture correction.

In conclusion, the axis 1 and axis 2 classification system for TMDs facilitates a comprehensive diagnostic approach, allowing healthcare providers to address the multifaceted nature of these disorders. By considering both the physical and psychosocial dimensions of TMDs, clinicians can develop more effective, personalized treatment plans, ultimately improving outcomes for patients suffering from these complex conditions.

### Limitations of this study

In this meta-analysis, only symptoms such as headache and internal damage of TMJ and not the complexity of TMD symptoms were considered in the studies only. Only Alrashdan’s study evaluated all types of TMD symptoms by comparing Axis I with Axis II. Therefore the results of the meta-analysis show high heterogeneity for this reason. Also, there could be, as shown in the various studies, some symptoms more associated with the psychological aspect and other symptoms less associated with the psychological aspect. In these cases, Axis II would give false negatives. Therefore, more studies taking into account single symptomatology are needed to correlate Axis I with Axis II effectively.

## Conclusion

In conclusion, DC/TMD is an effective tool for diagnosing TMD. It improves the accuracy of TMD diagnosis, allows for the classification of subtypes, and assesses psychosocial factors that may impact the development or maintenance of TMD symptoms. By comprehensively setting physical and psychosocial factors, DC/TMD can lead to a more targeted and effective treatment plan, improving patient outcomes.

## Data Availability

The data will be available on reasonable request from the corresponding author.
